# Reprogramming of Lipid Metabolism in Lung Cancer: An Overview with Focus on EGFR-Mutated Non-Small Cell Lung Cancer

**DOI:** 10.3390/cells11030413

**Published:** 2022-01-25

**Authors:** Kamal Eltayeb, Silvia La Monica, Marcello Tiseo, Roberta Alfieri, Claudia Fumarola

**Affiliations:** 1 Department of Medicine and Surgery, University of Parma, 43126 Parma, Italy; kamal.eltayebelemammohamedahmed@unipr.it (K.E.); marcello.tiseo@unipr.it (M.T.); roberta.alfieri@unipr.it (R.A.); claudia.fumarola@unipr.it (C.F.); 2 Medical Oncology Unit, University Hospital of Parma, 43126 Parma, Italy

**Keywords:** lipid metabolism, lung cancer, EGFR, EGFR-TKI resistance

## Abstract

Lung cancer is the leading cause of cancer deaths worldwide. Most of lung cancer cases are classified as non-small cell lung cancers (NSCLC). EGFR has become an important therapeutic target for the treatment of NSCLC patients, and inhibitors targeting the kinase domain of EGFR are currently used in clinical settings. Recently, an increasing interest has emerged toward understanding the mechanisms and biological consequences associated with lipid reprogramming in cancer. Increased uptake, synthesis, oxidation, or storage of lipids has been demonstrated to contribute to the growth of many types of cancer, including lung cancer. In this review, we provide an overview of metabolism in cancer and then explore in more detail the role of lipid metabolic reprogramming in lung cancer development and progression and in resistance to therapies, emphasizing its connection with EGFR signaling. In addition, we summarize the potential therapeutic approaches targeting lipid metabolism for lung cancer treatment.

## 1. Introduction

Lung cancer is one of the most common cancers globally; it accounts for about 13% of all cancer diagnoses worldwide [1,2] and is the leading cause of death in both sexes [3]. It has a poor prognosis due to difficulties in early diagnosis [4]. Most lung cancers are detected at advanced stages, making them difficult to manage effectively [5]. Of lung tumors, 80% are classified as non-small cell lung cancers (NSCLC), about 10–15% are small cell lung cancers (SCLC), and 5% are lung carcinoid tumors [2,6,7]. The epidermal growth factor receptor (EGFR) plays a fundamental role in carcinogenesis and progression in many cancers. In NSCLC, dysregulation in EGFR signaling can be associated with *EGFR* overexpression or *EGFR*-activating mutations (exon 19 deletion and L858R point mutation are present in 10–20% of NSCLC Caucasian patients and in 50% of Asian patients). In the presence of *EGFR* oncogenic mutations, the use of EGFR tyrosine kinase inhibitors (TKIs) has revolutionized the treatment of NSCLC and, at present, osimertinib, the third-generation EGFR-TKI, is the first-line therapy for NSCLC *EGFR*-mutated patients. *KRAS* mutations represent another definite oncogenic driver in advanced NSCLC, occurring in about 20–30% of patients, and are mutually exclusive with *EGFR* mutations [8].

One of the main alterations that takes place within cancer cells is the reprogramming of metabolism. Cancer cells modify their metabolic pathways of carbohydrates, amino acids, and lipids to sustain their growth and proliferation and survive under a lack or shortage of nutrients. These changes allow them to meet the requirements of the rapid cell division and to avoid signals that would lead to their growth arrest and execution, such as apoptotic signals [7,9,10]. Among the metabolic alterations observed in lung cancer, those associated with lipid metabolism have recently received increasing attention and recognition [11]. In this review, we explored the role of lipid metabolic rewiring in lung cancer development and progression and in resistance to therapies, focusing in particular on its connection with EGFR signaling. In addition, we shed light on the potential strategies exploiting lipid metabolism as a therapeutic target for lung cancer treatment.

## 2. Metabolic Alterations in Cancer

Glucose is one of the crucial nutrients used as a rapid source of energy by cancer cells. In normal cells, glucose breakdown (glycolysis) takes place in the cytosol, producing pyruvate. The consumption and fate of pyruvate depend on the physiological state of the cells. In the presence of oxygen (aerobic glycolysis), pyruvate is transported to mitochondria to be oxidized in the tri-carboxylic acid (TCA) cycle. However, in case of oxygen shortage, pyruvate is converted to lactate in the cytosol to regenerate the oxidizing equivalent nicotine amide adenine dinucleotide (NAD) that guarantees continuous glucose oxidation in the absence of oxygen.

In tumor cells, glucose uptake is greatly increased, and pyruvate is converted to lactate regardless of oxygen availability or scarcity: a process called the “Warburg effect”, having been described for the first time by Otto Warburg in the 1920s [12,13]. In addition, tumor cells reprogram many other pathways that emanate from glycolysis, the description of which is out of the scope of this review.

Aside from alterations in glucose metabolism, the altered metabolism of glutamine is also frequently involved in cancer metabolic reprogramming. Glutamine is important for cancer growth, serving as a source of both carbon and nitrogen. Cancer cells exhibit increased glutaminolysis, converting glutamine to glutamate and ammonia. Glutamate is converted into alpha-ketoglutarate (α-KG) through transamination or oxidative deamination; α-KG is transported to the Krebs cycle to compensate metabolic intermediates consumed for biosynthetic processes and to generate reducing equivalent NADPH and fatty acids [14]. Glutamate is also used as a precursor of the antioxidant glutathione and is a source of amino groups for nonessential amino acids, such as serine and glycine, which are required for macromolecular synthesis. Glutamine can directly provide nitrogen for nucleotide synthesis, and a metabolic shift toward this biosynthetic pathway has been observed during malignant progression [15].

**Figure 1:**
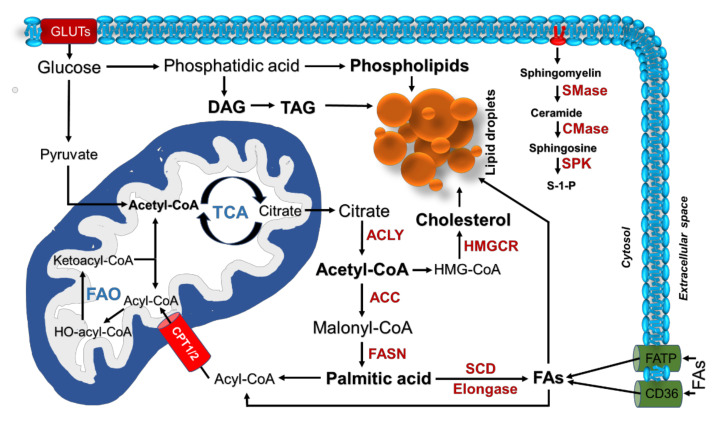
Lipid metabolism. Endogenous lipid synthesis is mediated by the accumulation of citrate in the cytosol and activation of the key lipogenic enzymes. Lipids (palmitate, cholesterol, DAG, MAG, and phospholipids) participate in multiple functions within the cell or are stored in lipid droplets. Sphingolipids, such as sphingomyelin, are converted to ceramide by sphingomyelinase, and ceramide is then converted into sphingosine-1-phosphate (S-1-P), which is involved in regulating many cellular functions. Exogenous uptake of fatty acids is mediated by different transporters (FATP and CD36). Fatty acid oxidation is mediated by transport of FAs into mitochondria through CPT1/2. Abbreviations: GLUTs: glucose transporters, TCA: tricarboxylic acid cycle, FAO: fatty acid oxidation, DAG: diacylglycerol, TAG: triacylglycerol, ACLY: ATP citrate lyase, ACC: acetyl-CoA carboxylase, FASN: fatty acid synthase, SCD: stearoyl-CoA desaturase, FAs: fatty acids, HMGCR: 3-hydroxy-3-methylglutaryl coenzyme A reductase, SMase: sphingomyelinase, CMase: ceramidase, SPK: sphingosine kinase, FATP: fatty acid transport protein, CPT1/2: carnitine palmitoyl transferase 1/2.

More recently, an increasing interest has emerged in understanding the role of lipid metabolism (Figure 1) in cancer. Lipids are a group of hydrophobic and amphipathic organic molecules categorized into simple, complex, and derived lipids. They are involved in cell structures, functions, and signaling. Cancer cells show increased demand for lipids to meet the immediate needs for membrane synthesis and energy production during division and to maintain the synthesis of signaling and bioactive molecules that sustain the cellular processes involved in cancer cell growth and metastasis [16].

Increased uptake and storage of lipids and enhanced expression of the proteins and enzymes involved in their metabolism occur in a variety of cancers. In addition, cancer cells synthesize lipids, in particular phospholipids (PLs), and cholesterol at higher rates than normal cells by increasing the expression level and activity of key enzymes along their biosynthetic pathways [7].

Most lipids are synthesized from fatty acids (FAs), a class of molecules consisting of hydrocarbon chains of different lengths and levels of desaturation, the metabolism of which is altered in cancer at multiple levels, including uptake, synthesis, and degradation. Fatty acid translocase (FAT)/CD36, a multifunctional protein that mediates FAs uptake is highly expressed in glioblastoma, oral squamous cell carcinoma, ovarian, and gastric cancer [17]. In contrast with normal cells, which preferentially obtain FAs from the outer environment, cancer cells also enhance their de novo synthesis, independently of the level of circulating lipids.

FA synthesis takes place in the cytosol from acetyl-CoA (AcCoA). AcCoA is synthesized through the breakdown of citrate by the action of the ATP citrate lyase (ACLY) enzyme. AcCoA is then carboxylated into malonyl-CoA (MaCoA) under the action of AcCoA carboxylase (ACC1/2); after that, it is consumed by the enzyme fatty acid synthase (FASN) to produce a 16C-long FA, palmitic acid. Palmitic acid (saturated fatty acid, SFA) undergoes either elongation through an elongase system to produce long-chain fatty acids (LCFAs) or desaturation by stearoyl-CoA desaturase-1 (SCD1). LCFAs can also be desaturated into mono-unsaturated fatty acids (MUFAs) or poly-unsaturated fatty acids (PUFAs). SFAs or MUFAs can be further used for the synthesis of neutral lipids, such as triglycerides (TGs), and stored in organelles termed ‘lipid droplets’ (LDs).

Enzymes involved in FA synthesis are highly expressed in many types of cancer, and their pharmacological inhibition has been shown to exert anti-cancer activity [18]. ACLY and FASN upregulation has been shown in colorectal, gastric, breast, liver, and lung cancer, and their overexpression has been significantly associated with poor survival in lung cancer patients [12,16]. SCD1 has been demonstrated to play a crucial role in cancer development and progression, promoting malignant transformation [19]. In addition, excess lipids are stored in LDs, resulting in a significant increase in LDs in many cancer cell types, including lung cancer cells, compared with normal cells [20]. It is worth noting that, in addition to having a role in lipid storage, LDs have important roles in energy metabolism regulation, cell proliferation and survival, immune response, signal transduction, and other processes relevant for cancer progression [21].

The incorporation of FAs into phospholipids and triglycerides needs the activation of FAs to fatty acyl-CoA, a reaction catalyzed by the enzyme fatty acyl-CoA synthetase (ACS). Long-chain ACSs (ACSLs), the enzymes responsible for the activation of the most abundant long-chain FAs, are frequently deregulated in cancer [22]. It is worth noting that FAs activation by ACS also represents the initial step required for mitochondrial FA β-oxidation (FAO), a multi-step process that allows for the breakdown of FAs into acetyl-CoA, which then enters the TCA to aid ATP generation. Multiple lines of evidence indicate that many cancer cells reprogram FAO and rely on this process for proliferation, survival, drug resistance, or metastasis [23]. Dysregulation of FAO in cancer has been correlated with the overexpression of various FAO enzymes; among them, a relevant role in cancer metabolic rewiring is attributed to carnitine palmitoyltransferase 1 (CPT1), the key rate-limiting enzyme of FAO that is responsible for the transfer of FAs from cytosol to mitochondria (Figure 1) [20]. For instance, CPT1A overexpression has been shown to correlate with poor patient outcomes of acute myeloid leukemia (AML) and ovarian cancer [23]. In addition, an increased expression of CPT1C associated with increased FA oxidation has been found in tumor cells more resistant to metabolic stress such as glucose deprivation [24].

It is well recognized that cancer metabolic reprogramming depends on the activation of oncogenic signaling transduction pathways and on the loss of tumor suppressors, which result in the upregulation of proteins and enzymes involved in the transport and metabolism of glucose, glutamine, or lipids [25,26].

**Figure 2:**
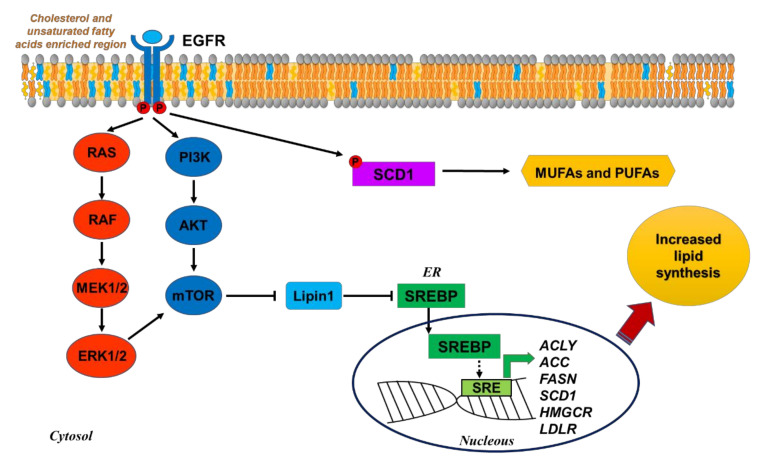
Modulation of lipid metabolism by EGFR signaling. EGFR induces lipogenesis activating the PI3K/AKT and MAPK pathways, which converge on mTOR-dependent inhibition of Lipin1. This event allows the translocation of SREBP into the nucleus. SREBP acts as a transcription factor for different genes involved in lipid metabolism, such as *ACLY*, *ACC*, *FASN*, *SCD1* (FA synthesis), *HMGCR*, and *LDLR* (cholesterol synthesis and uptake). EGFR directly phosphorylates SCD1 at tyrosine 55 and promotes its stabilization, increasing MUFAs production. MUFAs and cholesterol contribute to the activation of EGFR signaling through modulating cell membrane stability and fluidity.

EGFR, phosphatidylinositol 3-kinase (PI3K)/AKT, and RAS/MAPK signaling are key regulators of metabolism, including lipid metabolism, in cancer (Figure 2).

The PI3K/AKT pathway promotes de novo lipid synthesis through both transcriptional and post-translational mechanisms. AKT can directly phosphorylate and activate ACLY, stimulating the production of the cytosolic acetyl-CoA required for sterol and FA synthesis [27]. In addition, PI3K/AKT, as well as RAS/MAPK, pathways modulate the expression of genes involved in FA synthesis, such as FASN, through mTORC1-dependent activation of the sterol regulatory element-binding protein-1 (SREBP1) transcription factor [28,29]. In particular, mTORC1 inhibits Lipin1, a negative regulator of SREBP1. SREBP1 belongs to a family of transcription factors (SREBP1-a, SREBP1-c, and SREBP2) that plays a central role in the regulation of lipid metabolism and is upregulated in multiple cancers [30]. SREBP1 primarily regulates FA synthesis and phospholipid and triacylglycerol (TAG) synthesis, while SREBP2 is a master regulator of cholesterol synthesis [25,30]. Aside from upregulating SREBP1, the PI3K/AKT pathway has been shown to modulate the expression of SREBP2, although with effects that are context-dependent [31].

In a recent study, it has been demonstrated that SREBP1 cooperates with myc to activate FA synthesis, contributing to the growth of myc-driven cancer in animal models [32]. However, myc has been found bound to the promoters of FA synthesis genes, such as the *FASN* and *SCD* genes, suggesting that it can also stimulate FA synthesis independently of SREBP1 [33]. Myc can also enhance the mitochondrial synthesis of acetyl-CoA and the de novo synthesis of palmitate [34] and has been shown to play a role in the regulation of FAO [23].

Other important players involved in lipid metabolic reprogramming in cancer are the Hippo, Wnt/β-catenin, and JAK/STAT3 signaling pathways [35,36].

In addition to the oncogenic-dependent modulation, lipid metabolic reprogramming in cancer cells is greatly affected by the conditions of the tumor microenvironment (TME), such as hypoxia or acidic pH, or by signals coming from other cells in the TME, including immune cells, adipocytes, endothelial cells, and fibroblasts [37].

## 3. Alterations of Lipid Metabolism in Lung Cancer

### 3.1. Fatty Acid Biosynthesis

Many studies have demonstrated the role of altered FA biosynthesis in the progression of lung cancer. Phospholipid, neutral lipid, and triglyceride levels have also been demonstrated to be increased compared to normal lung tissues due to the enhanced expression of enzymes involved in their synthesis [38], such as ACLY, ACC, FASN, and SCD1.

Migita et al. [39] found that phosphorylated *ACLY* was overexpressed in 162 lung adenocarcinoma tumors and showed a correlation with stage, differentiation grade, and poorer prognosis. Accordingly, they also found a correlation between p-ACLY and p-AKT in NSCLC cell lines and demonstrated that ACLY phosphorylation was directly mediated by AKT. 

Moreover, inhibition of ACLY, either pharmacologically or through RNAi, resulted in cell cycle arrest and apoptosis induction in lung cancer cells in vitro and in vivo as its inhibition led to depleted levels of AcCoA, which resulted in FA synthesis reduction [40].

Xin et al. [41] demonstrated that the expression of *ACLY* is negatively regulated by miRNA-22 in different tumor cell types, including lung cancer. Treatment with miRNA-22, downregulating lipogenesis, attenuated lung cancer cell proliferation/invasion and promoted apoptosis in vitro and suppressed tumor growth and metastasis in mouse tumor xenografts. 

ACC1 and ACC2 isoforms were found to be overexpressed in lung cancer, and high expression levels of these enzymes were correlated with poor prognosis in NSCLC patients [42]. ACC1/2 promoted the growth and progression of NSCLC cells by enhancing FA synthesis both in vitro and in vivo, and their inhibition (by RNA interference or chemical inhibitors) led to growth arrest and activation of apoptotic signals [43].

Several studies reported that increased levels of FASN expression are detectable early during the development of lung cancer, particularly in patients with a prolonged history of smoking, and are correlated with aggressiveness in stage I lung carcinomas [44,45,46].

The upregulation of FASN in lung cancer has been associated with the overexpression of B7-H3, a glycoprotein with immunoregulatory functions identified as a predictor of poor survival in NSCLC patients [47]. Mechanistically, B7-H3 was found to regulate FASN and, hence, lipogenesis by modulating the expression of SREBP-1 in NSCLC cell lines.

FASN was also reported to play a role in the acquisition of resistance to EGFR TKIs in EGFR-mutated NSCLC; the underlying mechanism involved an FASN-mediated palmitoylation of mutant EGFR, which conferred persistent activity to the receptor. In particular, Orlistat, an FDA-approved anti-obesity drug, inhibited FASN, blocked EGFR palmitoylation, and reduced survival of PC-9GR and H1975 gefitinib-resistant cells [48]. In another study analyzing FASN expression in tissues from NSCLC patients before and after EGFR-TKI treatment, increased FASN levels were detected in relapsed tissues (after TKI treatment) compared to tissues before the treatment. In addition, in some unresponsive patients, after TKI treatment, the expression of FASN was significantly higher compared to those who responded, suggesting that elevated FASN may contribute to a decreased response to EGFR-TKI treatment [49]. The same study provided a mechanistic explanation for these observations, demonstrating a connection between the upregulation of the SREBP1/ACC/FASN axis and resistance to osimertinib. In sensitive cells, osimertinib promoted GSK3/FBXW7-mediated degradation of the mature form of SREBP1, significantly decreasing the levels of SREBP1 targets, FASN and ACC, and reducing the content of LDs. This mechanism was lost in osimertinib-resistant cells, which showed elevated levels of SREBP1, FASN, and ACC and sustained lipid metabolism. Sensitivity to EGFR inhibition was restored by genetic knockdown of *SREBP1* in vitro and in vivo, suggesting SREBP1/ACC/FASN targeting as a valuable strategy to overcome acquired resistance to EGFR TKIs. Moreover, Xu et al. [50] showed that the constitutive SREBP1-mediated activation of the lipogenic pathway is a key mediator of gefitinib-acquired resistance in EGFR mutant lung cancer, and targeting SREBP1 with fatostatin restored gefitinib sensitivity in resistant NSCLC in both in vitro and in vivo models. 

In another study investigating the role of FASN in NSCLC, it was demonstrated that the stable suppression of FASN by using lentiviral FASN small hairpin RNA in NSCLC cell lines had significant growth inhibitory effects both in vitro and in mouse xenografts. Interestingly, FASN deficiency impaired glucose metabolism through downregulation of AKT and ERK signaling, significantly reducing ATP and lactate production [51], a finding that underlies the interconnection among different metabolic routes in cancer.

FASN targeting was also evaluated as a strategy to improve sensitivity to radiotherapy in NSCLC. In particular, knockdown of *FASN* increased radiosensitivity in A549 NSCLC cells by reducing the expression levels of DNA double-strand break (DSB) repair-associated proteins and enhancing cell cycle arrest at the G2/M phase and apoptosis [52].

Analyzing a large cohort of lung adenocarcinoma, Huang et al. [53] demonstrated that expression of SCD1 is increased in more than 50% of cancer tissues compared to adjacent tissues and, in contrast with FASN expression, correlates with a later cancer stage.

SCD1 was shown to play a remarkable role in the survival and propagation of lung cancer stem cells (CSCs) [54]. In three-dimensional (3D) cultures from human lung adenocarcinoma, SCD1 was demonstrated to control the Hippo pathway by modulating the activity of its main effectors, Yes-associated protein (YAP) and transcriptional co-activator with a PDZ-binding motif (TAZ), an effect dependent on the activation of the Wnt/β-catenin pathway [55]. Accordingly, the high co-expression levels of SCD1, β-catenin, and YAP/TAZ emerged as a strong negative prognostic marker in lung adenocarcinoma.

Evidence indicates that SCD1 activates PI3K/AKT and RAS/ERK pathways. A study conducted by Nashed et al. [56] demonstrated that inhibition of SCD1 in NSCLC cancer cells negatively affected the phosphorylation of EGFR upon ligand binding, leading to the inactivation of ERK, AKT, and mTOR signaling cascades, thus, causing a defective mitogenic response to EGF. This effect was attributed to changes in the surroundings of the EGF receptors, specifically to alterations of the lateral mobility of plasma lipid microdomains. Moreover, the authors observed potentiated growth inhibitory effects of the first-generation EGFR inhibitor gefitinib upon treating lung cancer cells with SCD1 blockers. This study provided a clue for the possible enhancement of the anti-tumor activity of EGFR inhibitors through blocking SCD1 activity.

In another study [57], it was shown that PUFAs with longer chain lengths, such as FA(20:5) and FA(22:6), were significantly higher in lung pleural effusions from *EGFR* mutant NSCLC patients compared to non-*EGFR* mutant, suggesting that they may play a role in sustaining the activation of mutant *EGFR*.

Interestingly, aside from SCD1-dependent modulation of EGFR signaling, an inverse regulation has also been demonstrated, in which EGFR directly phosphorylates SCD1 at tyrosine 55 and promotes its stabilization, thereby increasing MUFAs production [19] (Figure 2).

The role of SCD1 in lung cancer and its link with AKT signaling also emerged from a study conducted by Scaglia and Igal. Knockdown of the *SCD1* gene in A549 NSCLC cells decreased the MUFA/SFA ratio and reduced glucose incorporation into cell lipids, with consequent impairment of cell proliferation and anchorage-independent growth associated with induction of apoptosis. These effects were associated with the dephosphorylation of AKT and its downstream effector glycogen synthase kinase-3β (GSK-3β). Interestingly, the addition of exogenous oleic acid to the cells to provide a high lipid environment neither reversed the blockage of AKT activation nor rescued the cells from growth arrest and apoptosis, suggesting the relevance of the endogenous synthesis of MUFAs for proliferation and survival of NSCLC cells. The role of SCD1 in NSCLC cell growth was also confirmed in tumor xenografts in mice [58].

### 3.2. Transport and Storage of Lipids

Despite the central role of lipid synthesis inside tumors, some cancer types increase lipid uptake from surroundings. In NSCLC, membrane proteins involved in this process are found to be overexpressed and play a remarkable role in cancer progression.

A study evaluating the role of miRNA-21 in regulating FA metabolism in NSCLC cell lines demonstrated that miRNA-21 mimic treatment increased cell proliferation and migration by inducing the expression level of CD36. On the other hand, downregulation of CD36 reduced the intracellular lipid contents and inhibited miRNA-21-mediated effect on tumor cell growth. These findings demonstrated the contribution of the miRNA-21/CD36 axis to NSCLC cell growth through modulation of FAs uptake and metabolism [59].

FA-binding proteins (FABPs) are also involved in the transport of lipids inside the cell and have been proved to play a remarkable role in tumorigenesis [60]. Tang et al. [61] studied the expression of FABP3 and FABP4 in NSCLC patients and found a significant increase of their mRNA expression in cancerous tissues compared to non-cancerous; high levels of FABP3 or FABP4 expression were associated with advanced tumor node metastasis stage and negatively affected the overall survival of NSCLC patients.

Recently, it was found that NSCLC tumor tissues had a higher content of LDs compared with pericancer tissues, which was further increased in tumor tissues of patients who underwent EGFR-TKI treatment [62]. In addition, gefitinib-resistant, *EGFR*-mutated NSCLC cells showed a higher content of LDs compared to the parental gefitinib-sensitive PC9 and HCC827 cells, suggesting a role of LD accumulation in EGFR-TKI resistance. Interestingly, reducing LD formation by pharmacological inhibition of SCD1 could reverse gefitinib-resistance, promoting inhibition of tumor growth and inducing apoptosis in vitro and in vivo [62].

Since LDs negatively regulate intracellular reactive oxygen species (ROS) levels and reduced ROS levels can promote EGFR phosphorylation, it has been hypothesized that LD accumulation, by regulating cellular ROS levels, contributes to EGFR activation, suggesting the use of LD inhibitors to interfere with EGFR activation [21].

### 3.3. Fatty Acid Oxidation

A metabolomic analysis performed to investigate the metabolic alterations in lung cancer demonstrated an increased content of metabolites associated with FAO, such as acylcarnitines, in SCLC cells as compared with NSCLC cells and normal epithelial cells. Accordingly, SCLC cells expressed higher levels of CPT1, the key enzyme located in mitochondria for FAO, indicating that FAO is upregulated in this subtype of lung cancer [63].

Human lung cancers were demonstrated to also express high levels of ACSL3, one of the enzymes responsible for the conversion of FAs into acyl-CoA esters, the substrates for β-oxidation and lipid synthesis [64].

In mutant *KRAS* NSCLC cells, ACSL3 promoted the accumulation of neutral lipids in LDs, presumably to provide substrates to fuel β-oxidation under energy stress conditions.

*ACSL3* silencing induced cell death in vitro by depleting cellular ATP and impaired tumor growth in vivo, suggesting the involvement of this enzyme in mutant *KRAS* lung cancer growth [64].

It was reported that CPT1C, a brain-specific metabolic enzyme, is frequently upregulated in human lung tumors and confers resistance to metabolic stress, such as glucose deprivation and hypoxia, by increasing FAO to sustain ATP production [24].

Under energy stress, FAO also becomes the major source of the reducing equivalent NADPH required to maintain the redox balance. In these conditions, cancer cells increase FAO through the AMPK–ACC axis [65].

It is worth noting that the role of FAO as a modulator of redox homeostasis during cancer progression is context-dependent.

In mutant *KRAS* lung adenocarcinoma mouse models, constitutive deficiency of LKB1, a known upstream regulator of AMPK, was associated with impairment of FAO, due to inactivation of the AMPK–ACC axis and ROS accumulation. Increased ROS promoted adenocarcinoma to squamous cell carcinoma transdifferentiation, allowing a metabolic adaptation that may contribute to cancer progression and influence the response to metabolic-targeted therapies [66].

A metabolic shift toward FAO, associated with the upregulation of antioxidant gene programs, has been reported in cycling cells emerging in EGFR mutant PC9 NSCLC cell cultures during treatment with osimertinib [67].

These cells, defined as cycling persisters, arose from a pre-existing cell lineage, and their proliferation under drug pressure might contribute to the emergence of drug resistance. Interestingly, overexpressing CPT1A in PC9 cells significantly increased the fraction of cycling persisters after 14 days of osimertinib treatment. The opposite effect was observed when the cells were treated with the CPT1 inhibitor etomoxir.

Accordingly, cell RNA-seq profiles from human *EGFR* mutant lung adenocarcinomas tumor samples demonstrated that FA metabolism and ROS pathway signatures were gradually increased during treatment from naïve to resistant relapsed tumors [67].

In addition, FAO promotes intermediates supporting cellular growth of lung cancer cells resistant to radiation and paclitaxel [68,69].

Other aspects of lipid metabolic reprogramming in lung cancer include alterations in the metabolism of sphingolipids and cholesterol.

### 3.4. Sphingolipid Metabolism

Sphingolipids, in addition to their structural role in cell membranes, are important for the growth, proliferation, survival, migration, invasion, and metastasis of cancer cells. Ceramide is composed of a sphingosine base and amide-linked acyl chains varying in length from C14 to C26 and is the central molecule of sphingolipid metabolism. Indeed, ceramide is the precursor of complex sphingolipids, such as sphingosine-1-phosphate (S1P), sphingomyelin, ceramide-1-phosphate, and glucosylceramide (GlcCer). It is worth noting that ceramides may have pro- or anti-tumor functions, and these opposing activities depend on their fatty acid chain lengths, subcellular localization, and/or direct downstream targets [70]. Different studies reported increased levels of ceramide in smoker patients with emphysema who are susceptible to develop lung cancer [71]. It has been demonstrated that cigarette smoking induces the activation of two signaling pathways: one activates sphingomyelinase, the enzyme responsible for sphingomyelin hydrolysis to ceramide, and the other activates EGFR, leading to sustained proliferative signaling. These pathways cooperate and interact, allowing the localization of EGFR in ceramide-enriched regions of the plasma membrane, a finding that supports the role of sphingomyelinase/ceramide in the tumorigenic signaling induced by aberrant activation of EGFR [71].

Gene expression analysis of NSCLC tissues and lung cancer cell lines revealed an increased expression of ceramide synthase gene (*CERS6*). Overexpression of this gene correlated with poor prognosis and lymph node metastasis in NSCLC patients. Silencing of *CERS6* decreased ceramide production, which resulted in reduced cell migration in vitro. Moreover, knockdown of the *CERS6* gene attenuated the metastatic potential of lung cancer cells in mice, supporting the relevance of ceramide in lung cancer migration and metastasis [72].

Another enzyme of sphingolipid metabolism that has been correlated with poor survival in NSCLC is sphingosine kinase 2 (SPHK2), which synthesizes S1P by phosphorylating sphingosine. Expression of this enzyme was also associated with resistance to gefitinib EGFR TKI. The relevance of SPHK2 in lung cancer cell growth was confirmed in preclinical experiments demonstrating that pharmacological inhibition by ABC294640 of this enzyme led to cell cycle arrest, increased apoptosis, and suppression of tumor growth in vitro and in vivo [73].

According to these findings, higher concentrations of S1P in plasma were associated with increased future risk of lung cancer development [74].

Other ceramide derivatives that have received attention for their contribution to cancer progression are GlcCer and glycosphingolipids, which are generated through glycosylation of ceramide by glucosylceramide synthase (GCS). A higher content of glycosylated ceramide and sphingomyelin species was observed in malignant lung pleural effusions from NSCLC patients compared to benign lung pleural effusions [57]. The glycosphingolipid GM2 was shown to induce invasive properties via epithelial mesenchymal transition (EMT) in lung cancer cells and was found overexpressed in invasive irradiation-tolerant lung cancer cells, suggesting that its inhibition may serve as a potential approach for lung cancer treatment after radiotherapy [75].

An increased production of GlcCer has been proposed as an adaptive response to evade apoptosis by reducing the intracellular content of ceramide [76]. Indeed, ceramide is a well-known regulator of apoptosis, and its accumulation has been reported to induce apoptosis in a variety of cancers, including lung cancer [77,78]. Cisplatin-acquired resistance in malignant pleural mesothelioma (MPM) and NSCLC cells was associated with increased cell surface expression of globotriaosylceramide (Gb3), which enhanced cell proliferation and blocked ceramide-induced apoptosis; treatment with GCS inhibitors, reducing ceramide glycosylation, was able to overcome cisplatin resistance. These findings suggest the cell surface expression of Gb3 as a potential biomarker of cisplatin resistance in MPM and NSCLC cancer [76].

Another study [79] demonstrating the pro-apoptotic role of ceramide in lung cancer showed that treatment of NSCLC H1299 cells with exogenous C8-ceramide promoted the switch of superoxide dismutase 1 (SOD1) to SOD2, which increased the endogenous levels of ROS, inhibiting cell proliferation and inducing apoptosis.

### 3.5. Cholesterol Metabolism

Cholesterol is essential for cell membrane integrity; it modulates its fluidity and rigidity, as well as other functions [13]. It is synthesized through the mevalonate pathway, and its role in cancer is under focus. HMG-CoA reductase (HMGCR) is the rate-limiting enzyme in the cholesterol synthesis pathway and is targeted by statins. The mevalonate and its metabolites are essential for cancer progression in different cancer types including prostate, liver, breast, and lung cancers [80].

Overexpression of squalene synthase (SQS), the enzyme that determines the switch of the mevalonate pathway toward sterol biosynthesis, has been associated with poor outcomes in lung cancer patients. In addition, SQS expression promoted the metastatic potential of lung cancer cells, and its pharmacological inhibition in metastatic CL1-5 lung cancer cells suppressed invasiveness in vitro and lung metastasis in vivo [81].

As previously mentioned, endogenous cholesterol, as well as FA synthesis, is controlled by the expression of enzymes regulated by SREBP. Li et al. [82] showed that SREBP inhibition by betulin, fatostatin, or 25-hydroxycholesterol in NSCLC cells downregulated the expression of *FASN*, *SCD*, and *HMGCR* genes, reducing the proportion of cholesterol and unsaturated FAs on the cell membrane. The consequent decrease of cell membrane fluidity reduced the phosphorylation and activation of EGFR, increasing cell sensitivity to gefitinib both in vitro and in vivo (Figure 2). These results demonstrate the role of SREBP inhibition in enhancing the therapeutic response of lung cancer cells to EGFR TKIs.

Moreover, LDLR (low-density lipoprotein receptor) was highly expressed in EGFR-mutated NSCLC cells, suggesting a role of EGFR signaling in upregulating LDLR expression through an AKT-mediated, SREBP1-dependent pathway [83].

Interestingly, in a murine model of KRAS-driven lung adenocarcinoma, it was demonstrated that myc plays a role in the modulation of cholesterol metabolism. In fact, increased myc activity reduced the efflux of cholesterol while enhancing its influx and the accumulation of cholesteryl esters in LDs. This mechanism was proposed to contribute to the growth of KRAS, myc-overexpressing NSCLC cancers, suggesting that targeting cholesterol metabolism may be explored as a novel approach for lung cancer treatment with diagnostic and stratification potential [84].

## 4. Targeting Lipid Metabolic Reprogramming in Lung Cancer

**Figure 3:**
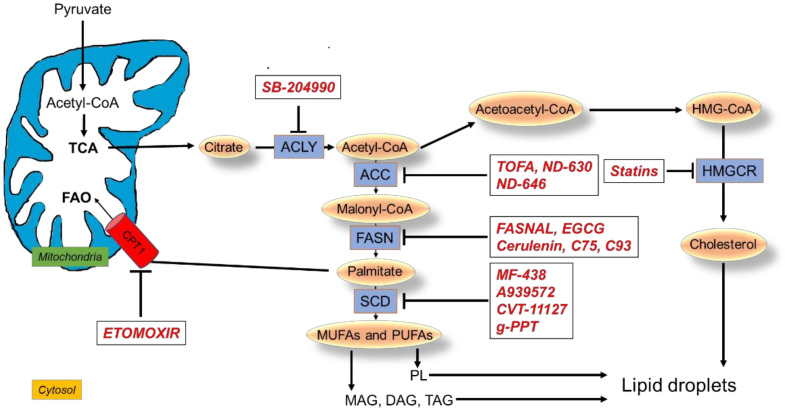
Targeting lipid metabolism in lung cancer. Inhibitors of lipid synthesis and oxidation used in preclinical studies in lung cancer models.

Multiple drugs targeting lipid metabolism have been developed and are being investigated for treatment of lung cancer (Figure 3).

### 4.1. Targeting FA Synthesis

#### 4.1.1. Inhibitors of ACLY

SB-204990: SB-204990 is an effective, potent, selective inhibitor of the ACLY enzyme that decreases cytosolic AcCoA [85]. SB-204990 was found to inhibit tumor growth of A549 NSCLC and PC3 prostate cancer cells both in vitro and in vivo [40].

It has been demonstrated that lung cancer cells increase the level of ACLY by decreasing its degradation. In particular, the ubiquitin-dependent degradation of ACLY is inhibited by the downregulation of a protein called Cullin3 (a tumor suppressor); this protein is essential for forming the ubiquitin ligase complex needed for this process. SB-204990 was found to abolish the promoting effect of Cullin3 downregulation on lipid synthesis, cell proliferation, and tumor growth, suggesting that targeting ACLY could be a potential therapeutic strategy for lung cancer with reduced expression of Cullin3 [86].

#### 4.1.2. Inhibitors of ACC

TOFA: 5-tetradecyloxy-2-furoic acid (TOFA) is an allosteric inhibitor of the ACC1 isoform. TOFA was found to exert cytotoxic effects in H460 NSCLC cells by blocking FA synthesis and inducing apoptosis in a dose-dependent manner; administration of exogenous palmitic acid prevented TOFA-induced apoptosis. These findings suggest ACC1 as a potential target for lung cancer intervention [87,88,89].

ND-630 and ND-646: ND-630 is an allosteric inhibitor of ACC1 and ACC2. It acts by inhibiting the allosteric protein–protein interaction, preventing the enzyme subunit’s dimerization by binding to the phosphopeptide acceptor and its dimerization site [90]. ND-646 is the amide derivative of ND-630 [91]. By inhibiting both enzymes and preventing the ability of ACC2 to compensate for ACC1 inhibition, ND-646 is a potent inhibitor of lipid synthesis. The gene for ACC1 was found highly expressed in NSCLC cell lines, and their treatment with ND-646 reduced the pools of FAs, inhibiting cell proliferation and inducing apoptosis. In addition, ND-646 suppressed tumor growth in a mouse model of NSCLC when administered alone or combined with the standard-of-care drug carboplatin, suggesting a potential utilization of the ACC inhibitors as anti-cancer therapy [91].

#### 4.1.3. Inhibitors of FASN

Fasnall and EGCG: Fasnall is a thiophenopyrimidine that selectively targets the enzyme FASN. In NSCLC autophagy-deficient cells, which are more dependent on FAO for energy production, fasnall exerted anti-proliferative activity and induced cell death [92].

Moreover, in A549 NSCLC cells, epigallocatechin gallate (EGCG), one of the polyphenolic compounds of green tea, reduced FASN activity and dephosphorylated EGFR and the AKT/mTOR and MAPK downstream signaling pathways with induction of apoptosis. The anti-cancer activity of this compound was confirmed in a mouse model of A549 lung cancer xenograft [93].

The EGCG derivative G28 showed promising results in overcoming the resistance to gefitinib and osimertinib in *EGFR*-mutated NSCLC TKI-resistant cell models expressing high FASN levels [94].

Inhibition of FASN with two novel galloyl esters of trans-stilbene led to the deactivation of AKT signaling and induction of apoptosis in NSCLC cells [95].

Cerulenin, C75, and C93: Cerulenin inhibits FASN by non-competitive binding to the enzyme: it binds to the active site of FASN, preventing the condensation reaction between AcCoA and malonyl-CoA [96]. Since cerulenin is chemically unstable, another small molecule inhibitor has been developed, C75. This molecule was demonstrated to inhibit FASN in a xenograft model of NSCLC cancer, significantly reducing tumor growth. However, it was also shown to increase FAO rate, being also a strong activator of CPT1 [93]. Considering that C75 showed relevant side effects [97], another compound was developed to inhibit FASN, C93, which promoted a remarkable growth reduction in NSCLC orthotopic xenograft tumors [96] and prevented tumor growth in carcinogen-exposed mice [45]. Importantly, inhibition of FASN by C93 did not induce FAO in H460 lung cancer cells at any dose level, compared to an approximate 80% increase in FAO upon treatment with C75 [98]. However, in A549 and H1975 human lung cancer cell lines, C93 treatment triggered a protective response mediated by NF-kB signaling activation, and the pharmacological inhibition of both NF-kB and FASN led to more effective cell killing than inhibiting either NF-kB or FASN individually [99].

#### 4.1.4. Inhibitors of SCD1

MF-438: MF-438 is a thiadiazole–pyridazine derivative inhibitor of SCD1 [100]. It was shown that MF-438 increased the apoptotic rate, reduced markers of staminality, and decreased the tumorigenic potential in tumor spheroids generated from human H460 cells [54]. In another study in lung adenocarcinoma cells, cisplatin induced the formation of larger tumor spheroids compared to control and upregulated lung CSC markers. Interestingly, inhibiting SCD1 activity with MF-438 suppressed cisplatin-mediated induction of CSC markers and decreased the formation of tumor spheroids by inducing apoptosis [101].

A939572: A939572 (4-(2-Chlorophenoxy)-*N*-[3-(methyl carbamoyl)phenyl]piperidine-1-carboxamide) is another small molecule SCD1 inhibitor [102]. In A549 and H1573 NSCLC cell lines, the overexpression of SCD1 activated the EGFR/PI3K/AKT signaling, promoted the acquisition of metastatic potential, and conferred resistance to gefitinib-induced apoptosis. A939572 treatment restored gefitinib sensitivity, decreasing the cell viability, abrogating the EMT phenotype, and impairing the ability to migrate and invade. A939572-mediated anti-tumor effects were confirmed in SCD-overexpressing NSCLC cells in vivo [103].

CVT-11127: The inhibition of SCD activity with the novel, potent, and specific inhibitor CVT-11127 reduced lipid synthesis, inducing cell cycle arrest and apoptosis in H460 NSCLC cells [104]. Moreover, in human lung and breast cancer cells, the inhibition of SCD1 with CVT-11127 promoted the activation of AMP-activated kinase (AMPK) and the reduction of ACC activity, suggesting that SCD1 may regulate FA biosynthesis by controlling the activation of AMPK, which, in turn, phosphorylates ACC-reducing lipogenesis. The concomitant inhibition of glucose-mediated lipogenesis suggests the requirement of SCD1 activity in controlling this process in cancer cells [105].

g-PPT: Inhibition of SCD1 expression by (20S)-protopanaxatriol (g-PPT) reduced intracellular LDs in gefitinib-resistant, *EGFR*-mutated NSCLC cells. The combination of gefitinib and g-PPT restored sensitivity to gefitinib, providing evidence for the role of lipid metabolic reprogramming in the resistance to EGFR inhibitors and suggesting the utility of monitoring these metabolic changes to predict resistance [62].

### 4.2. Targeting FAO

Etomoxir: Etomoxir inhibits CPT1, the enzyme that transports long-chain FAs into mitochondria. Etomoxir treatment enhanced the growth inhibitory effects of radiation in spheroids generated from lung adenocarcinoma H460 cells. In addition, the combined treatment downregulated the expression of *Ki-67* and *cyclin D1* (proliferation biomarkers), *CD44* (stemness biomarker), and *CPT1A* (β-oxidation biomarker) and significantly reduced the hypoxic regions compared to either treatment alone, a relevant finding considering that hypoxia in tumors is associated with resistance toward radiotherapy [106].

Accordingly, etomoxir significantly decreased the oxygen consumption rate and the DNA repair capability of lung cancer cells resistant to radiotherapy and restored their radiosensitivity, suggesting CPT1A as a potential target for the treatment of radiation-resistant lung cancer [68].

In addition, inhibition of FAO using etomoxir reversed the resistance of lung adenocarcinoma cells to paclitaxel [69].

### 4.3. Targeting Cholesterol Metabolism

Statins: As previously mentioned, statins are a group of compounds that inhibit HMG-CoA reductase. In terms of overall survival (OS), NSCLC cancer patients are more likely to benefit from statin use than SCLC patients, who were found to be less susceptible to statin effects [107]. A randomized phase II study (NCT 01441349) is testing the efficacy of irinotecan/cisplatin combined with simvastatin in SCLC patients. The enhanced response to statins may also depend on many factors such as cancer stage or the genetic profile of the patients. It has been found that NSCLC patients with stage 4 benefit more than those in stages 1 to 3 [108].

Other studies showed significant improvement in progression-free survival (PFS) and OS for NSCLC patients treated with the combination of statins and EGFR TKIs [109,110]. In addition, Han et al. found that simvastatin may improve the efficacy of gefitinib in gefitinib-resistant, non-small cell lung patients [111], and a recent cohort study from Taiwan has shown that the mortality was significantly lower in patients treated with EGFR TKI in the statins group compared with the non-statins group [112]. By contrast, there were no significant differences in the efficacy between afatinib and the combination of afatinib with simvastatin in patients with nonadenocarcinomatous NSCLC, which is unlikely to have *EGFR* mutations [113].

In *EGFR* mutant NSCLC cell lines, the treatment with statins was shown to augment the anti-tumor effects of ACLY inhibition; this effect was attributed to the inhibition of PI3K/AKT and MAPK pathways [114]. It was reported that lovastatin inhibited EGF-induced EGFR autophosphorylation and its downstream signaling, and the combined treatment of gefitinib with lovastatin induced synergistic anti-proliferative and pro-apoptotic effects in cancer cell lines, including NSCLC cells [115]. In addition, in A549 NSCLC cells, lovastatin induced apoptosis [116] and enhanced radiosensitization, inhibiting the pro-survival AKT pathway [117]. It was also demonstrated that lovastatin and fluvastatin suppressed AKT and MAPK pathways in lung cancer cells, preventing cell growth and promoting apoptosis in vitro and in vivo [118]. Similarly, another inhibitor, simvastatin, induced apoptotic cell death in *EGFR*-mutated, gefitinib-resistant H1975 NSCLC cells by upregulating the pro-apoptotic Bcl-2 family member BIM [119]. Moreover, atorvastatin enhanced the anti-proliferative effect of gefitinib and osimertinib in TKI-sensitive, *EGFR*-mutated NSCLC models and overcame EGFR gefitinib resistance in H1975 cells [83].

## 5. Conclusions and Perspectives

Lipid metabolic reprogramming has emerged as a relevant feature contributing to lung cancer development and progression.

A variety of inhibitors targeting lipid metabolism at multiple levels have been developed and have shown promising efficacy for lung cancer treatment, at least in preclinical studies.

However, it is becoming evident that targeting a single molecule or pathway involved in lipid metabolism may not be sufficient to achieve durable suppression of cancer cell growth.

Indeed, considering that both the uptake of exogenous lipids and endogenous lipid synthesis contribute to providing lipid sources in cancer cells, compensation from dietary lipids may impair the effectiveness of therapeutic strategies based on inhibition of FA or cholesterol synthesis.

In addition, metabolic plasticity may allow cancer cells to continue utilizing lipids thanks to the activation of alternative pathways as an escape mechanism. For example, lung cancer cells have been recently shown to use an unconventional FA desaturation pathway, based on the desaturation of palmitate to sapienate, as an alternative to the well-characterized SCD-dependent pathway to support membrane biosynthesis during proliferation. Only the combined inhibition of both pathways effectively impaired cancer cell growth in vitro and in vivo by suppressing the metabolic plasticity in FA desaturation. Accordingly, sapienate metabolism was found active in lung carcinoma from human patients [120].

Based on these considerations, combinatorial approaches targeting lipid metabolism at different levels may represent a superior therapeutic option for halting cancer cell growth.

Alternative, valuable strategies may include the combination of lipid metabolism-targeted drugs with other targeted therapies or with conventional chemotherapy and radiation. This may be relevant for overcoming drug-resistance mechanisms associated with lipid metabolic reprogramming. In this regard, several studies, previously mentioned and summarized in Table 1, have demonstrated that targeting FA synthesis, FAO, LD accumulation, or cholesterol metabolism can restore sensitivity to EGFR-TKI inhibitors in those lung cancers whose resistance is correlated with lipid metabolic changes.

As a final consideration, expanding the knowledge of lipid metabolism in lung cancer may not only help open avenues for novel therapeutic opportunities, but may also contribute to identifying new therapeutic biomarkers. In this regard, a very recent study demonstrated that a high number of mutations of the lipid metabolism in NSCLC patients correlated significantly with the PFS after treatment with immune checkpoint inhibitors (ICIs), indicating that the lipid metabolism status may serve as a predictive biomarker to help the selection of NSCLC patients who may benefit from ICIs therapy [121].

## Figures and Tables

**Table 1:** Lipid metabolism alterations in EGFR TKI-resistant NSCLC models.

Lipid Metabolism Alteration	EGFR-TKI	Overcoming Resistance	Refs
FASN-mediated palmitoylation of mutant EGFR	Gefitinib	Orlistat treatment	[48]
Elevated levels of SREBP1 and/or FASN	Osimertinib	Genetic knockdown of SREBP1	[49]
FASN overexpression	Gefitinib/ Osimertinib	G28 treatment	[94]
Constitutive activation of SREBP1	Gefitinib	Fatostatin treatment	[50]
High LD content and SCD1 expression	Gefitinib	Inhibition of LDs formation by g-PPT	[62]
FAO upregulation	Osimertinib	Etomoxir treatment	[67]
LDLR expression upregulation	Gefitinib	Atorvastatin treatment	[83]
Undefined	Gefitinib	Simvastatin treatment	[119]
